# Primary Prevention: No Associations of Strength and Cardiorespiratory Fitness Status With Arterial Stiffness in Young School Children

**DOI:** 10.3389/fped.2020.00175

**Published:** 2020-05-07

**Authors:** Hannah Kirchhuebel, Renate Oberhoffer, Birgit Böhm

**Affiliations:** Faculty of Sport and Health Sciences, Institute of Preventive Pediatrics, Technical University Munich, Munich, Germany

**Keywords:** arterial stiffness, pulse wave velocity, children, cardiorespiratory fitness, hand grip strength

## Abstract

Pulse wave velocity (PWV) and central systolic blood pressure (CSBP) are well-established biomarkers of arterial stiffness. Further, fitness is known to be an important protective factor in adults in respect of vascular stiffening. However, the association of both muscular and cardiorespiratory fitness (CRF) with arterial properties in younger individuals has been inconsistent. The aim of the present study was to investigate the relationship between anthropometric data, CRF, strength status, and arterial stiffness parameters in German primary school children. A total of 76 children, age 6–11 years (63.2% males) were examined. Peripheral systolic blood pressure (PSBP) [mmHg] and peripheral diastolic blood pressure (PDBP) [mmHg] and PWV [m/s] were measured non-invasively after 10 min of rest with the oscillometric cuff-based Mobil-O-Graph (IEM, Healthcare, Stolberg, Germany). CSBP [mmHg] was calculated using the ARCSolver Algorithm (Austrian Institute of Technology, Vienna, Austria) based on the recorded brachial pulse waves. CRF was measured using the validated Progressive Aerobic Cardiovascular Endurance run (PACER), also called shuttle-run test, for estimating maximal aerobic capacity (VO_2max_). Hand-grip strength as an indicator of overall muscle strength was determined with the Jamar Analog Hand Dynamometer. The results were recorded in kilograms [kg]. For more detailed analyses, the study group was divided into subcohorts, namely a risk group including children with abnormal blood pressure or high body weight, and a healthy subgroup. Healthy children showed a positive association between PWV and body mass index (BMI) (*p* = 0.016) and CSBP and BMI (*p* = 0.033), respectively. Hand-grip strength was positively associated with CSBP (left: *p* = 0.013, right: *p* = 0.015) and PWV (left: *p* = 0.008, right: *p* = 0.002), as well as the number of shuttle run rounds were positively correlated to PWV (*p* = 0.038) in the whole cohort. No significant association of converted VO2max with arterial PWV was found. The multivariate regression analysis explained 38.8% (*R*^2^ = 0.388) of the variance and the model was a significant predictor of PWV [*F*_(6, 29)_ = 3.060, *p* = 0.019], however, none of the integrated covariates (BMI, number of shuttle run rounds, VO_2max_, dominant hand-grip strength) contributed significantly to the model. The lack of associations between fitness, strength and arterial stiffness might be explained by the few harmful lifestyle factors influencing vascular changes in the first decade of life.

## Introduction

Cardiovascular diseases (CVDs) are the biggest cause of death in Europe (1). Cardiovascular dysfunctions are often related to atherosclerosis. Risk factors and risk behaviors accelerate the development of endothelial lesions and the progression of arterial wall transformations. There is increasing evidence that arterial wall transformations start at a very early age and that the atherosclerotic process and arterial stiffening commences in childhood (2, 3).

Arterial function can be measured as the pulse wave velocity (PWV) a well-validated method to measure arterial distensibility (4). PWV is influenced by the elasticity of large arteries. A surrogate for arterial stiffness is the central systolic blood pressure (CSBP) (5) which has been proven to be more important than peripheral BP in predicting CVDs (6, 7). Literature also reveals the importance of CSBP in childhood as it is seen as a consistent predictor for development of arterial stiffness at an early age (8).

Physical activity involving endurance and strength training seems to play an important role in preventive cardiology in children.

Studies in children (9, 10) have examined the relationship between cardiorespiratory fitness (CRF) and arterial distensibility. Besides the positive effects of healthy CRF (11), literature also demonstrated that resistance training may be effective for improving vascular health in adults and adolescents (12–14).

However, despite the promising results of strength training in adolescents, data are lacking regarding strength status and the attenuating effect on arterial stiffness in primary school children in Germany (aged 11 years and younger). Accordingly, the aim of the present paper is to analyse the association between hand-grip strength, CRF, anthropometrics, and arterial stiffness in young healthy children.

Based on the available data on this topic, it was hypothesized that higher CRF would be associated with lower arterial stiffness. Since scientific evidence showed a positive effect on arterial compliance related to strength training, it was further hypothesized that an interaction might exist in young children as well.

Possible sex differences, age differences, and children with cardiovascular risk factors were taken into account.

## Methods

### Study Design and Study Population

Within the Bavarian health initiative “Healthy.Living.Bavaria” the heart health community project “*Pressure down—Activity up”* was implemented in December 2017 at a South Bavarian community in Germany within a community-university-government partnership context. The heart health project promotes the importance of cardiovascular risk screening across generations in different settings. Next to the diagnosis of hypertension in the elderly, emphasis is being laid on early cardiovascular risk screening in primary school children, focusing on blood pressure, arterial stiffness, cardiorespiratory fitness and strengths. The present cross-sectional study presents data of the participating primary school children.

**Figure 1:**
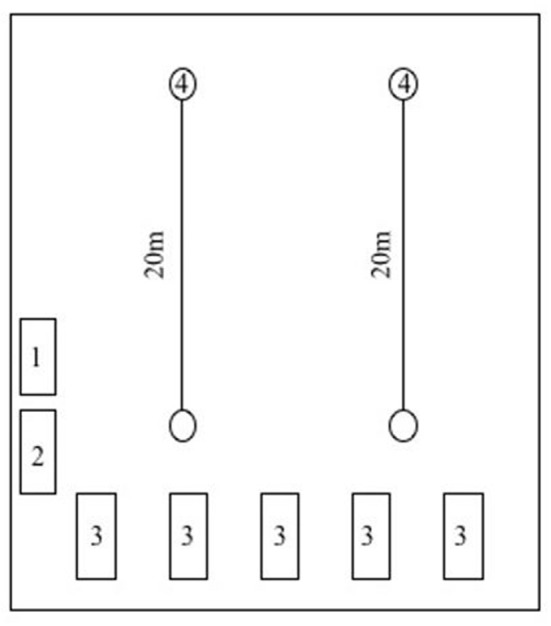
Study setting in school's gymnasium. Station 1: Measurement of body weight and height. Station 2: Measurement of hand grip strength. Station 3: Measurement of heart rate. blood pressure and pulse wave velocity. Station 4: Shuttle-run test.

The study was approved by the ethics committee of the Faculty of Medicine at the Technical University of Munich (162/18S) and was conducted in accordance with the principles of the Declaration of Helsinki. 110 children are attending the primary school from the 1st to 4th grade. Written informed consent was obtained from the children's guardians prior to the participation in the study, resulting in a study cohort of 76 primary school children, age 6–11 years (63.2% males). The children were recruited and measured in a school setting to make sure to reach apparently healthy children. Children entered the setting in groups of five, starting off with the individual assessment of body composition, measurement of blood pressure (PSBP, PDBP, CSBP, CDBP) and arterial stiffness (PWV), followed by the assessment of handgrip strength. Finally, the cardiorespiratory fitness was tested (Figure 1).

### Assessment of Body Composition

Anthropometric measurements were assessed by trained staff according to standardized guidelines (15). Portable scales (Withings Body Cardio) and a tape measure were used for quantifying body weight to the nearest 0.1 kg and height to the nearest 0.5 cm. Measurements of height and weight were performed without shoes and with the children wearing a single layer of light clothing over undergarments. The BMI was calculated as weight in kilograms divided by height in meters-squared and converted into z-scores using the reference values of a German cohort (16). According to the *German Obesity Association* childhood overweight was defined as a BMI between 90th and 97th percentile, obesity was defined as a BMI >the 97th percentile for children with the same age and sex. Underweight was defined as a BMI <10th percentile (16).

### Measurement of Blood Pressure and Pulse Wave Velocity

Peripheral blood pressure (PSBP and PDBP) and PWV were measured non-invasively after 10 min of rest with the oscillometric cuff-based Mobil-O-Graph (IEM, Healthcare, Stolberg, Germany). Peripheral blood pressure was defined in “Normotensive”: <90th percentile (z-score BP sysSDS (z)-score <1.282 or BP dia SDS (z)-score <1.282); “High normotensive” (90th−94th percentile) BP sys SDS (z)-score ≥ 1.282, <1.645, or BP dia SDS (z)-score ≥ 1.282, <1.645 and “Hypertensive Grade 1” (95th−99th percentile + 5 mmHg) BP sys SDS (z)-score ≥1.645 or BP dia SDS (z)-score ≥1.645 according to the recommendations of the European Society for Hypertension (17).

Central blood pressure (CSPB and CDBP) was calculated using the ARCSolver Algorithm (Austrian Institute of Technology, Vienna, Austria) based on the recorded brachial pulse waves (18). All tests were performed on the left upper arm with the patients in supine position. In order to select the appropriate arm cuff subjects' arm circumferences were assessed before starting the tests. The Mobil-O-Graph has already been validated for measurement of peripheral, central blood pressure and the analysis of pulse wave velocity (18, 19). Three measurements were performed and the mean of measurement two and three was then taken and classified according to German age and sex specific norm values (20).

### Assessment of Strength Status

Hand grip strength was measured using a *Jamar Analog Hand Dynamometer* with participants sitting in a straight-backed position with the feet flat on the floor. The shoulder was abducted and neutrally rotated, the elbow flexed at 90 degrees, and the forearm was in a neutral position. Participants were encouraged to keep the dynamometer vertically and in line with the forearm during the hand grip measurement trials to maintain the standard forearm and wrist positions. The arm was never supported by the examiner or by an armrest. The opposite arm was placed on the opposite thigh. This position has been well-documented as reliable (21, 22). Results were recorded in kilograms. All measurements were performed for both dominant and non-dominant hands. Subjects performed three maximum attempts for each measurement and the average value of these trials was recorded. Verbal encouragements at each trial were performed. The participants' hand grip strength data were displayed as left or right regardless of hand dominance.

### Assessment of Cardiorespiratory Fitness

Cardiorespiratory fitness was tested using the validated Progressive Aerobic Cardiovascular Endurance Run (PACER), also called shuttle-run, for estimating the maximal aerobic capacity (VO2max) (23). It was tested according to the Fitnessgram & Activitygram Test Manual (23). The children were encouraged to run as long as possible. Testing was stopped when the participant reached volitional fatigue or could not maintain the required pace for two consecutive shuttles. Subjects completed the test on average in groups of five people and if needed, a researcher ran alongside the participants to aid with pacing. Peak aerobic capacity was computed using the following formula: VO2max = 41.76799 + (0.49261 × PACER) − (0.00290 × PACER^2^) − (0.61613 × BMI) + (0.34787 × sex × age) (24), where PACER is the number of laps completed; for sex: 1 = boy and 0 = girl; and age is in years (24). Each child's VO2max value was then compared to sex- and age-specific standards.

### Data Analyses

All analyses were performed using SPSS version 23.0 software (IBM Corp, Armonk, NY). Data were tested for normal distribution using the Kolmogorov-Smirnoff test. Since data were skewed, all descriptive data were expressed in median values and interquartile ranges (Q1;Q3). The non- parametric Mann-Whitney-U test was calculated to assess differences between sex. Kruskal-Wallis test was used to evaluate differences between different age groups. Spearman's rho was applied to account for correlations between cardiovascular parameters, anthropometric measures, and fitness status. Effect size was calculated with the formula $r=\frac{z}{\surd n}$ (z = z-value, n = cohort size) (25). A multiple linear regression model was performed to find the strongest independent determinant on PWV and CSBP by including age, sex, BMI, dominant hand-grip strength, shuttle run rounds and VO_2max_. Diagnostics to avoid collinearity was carried out (variance influence factor <10.0). Normal distribution of residuals was proofed. For all analyses, a *p* < 0.05 was considered to be statistically significant.

## Results

### Descriptive Statistics of the Subject Characteristics

A total of 76 subjects (48 boys) were included in the analysis.

#### Sex Differences

In Table 1 anthropometric and cardiovascular data, CRF and hand grip strength testing results for the studied population and according to sex are displayed. No significant differences were observed for anthropometric measures and cardiovascular data between boys and girls. However, boys had a significantly higher cardiorespiratory fitness (50.3 ml/kg/min (47.2; 53.9) vs. 46.5 ml/kg/min (42.6; 49.5), *p* = 0.001). Effect size value was *r* = 0.365 and suggested a medium effect. Further, a trend of higher hand-grip results for boys was detected but not to a significant level. As no further differences were found between genders, analyses were continued by looking at the different age groups more closely.

**Table 1:** Subject characteristics according to sex.

	**Whole study group**				**Girls**				**Boys**				
**Variables**	***N***	**Median**	**25th**	**75th**	***N***	**Median**	**25th**	**75th**	***N***	**Median**	**25th**	**75th**	***p*-value**
**Anthropometrics**													
Age (years)	76	8.00	7.00	9.00	28	8.00	7.00	9.00	48	8.00	7.00	9.00	0.850
Height (cm)	76	132.50	127.00	142.00	28	131.00	127.00	139.50	48	133.00	128.00	142.00	0.428
Weight (kg)	76	28.00	25.00	34.00	28	26.70	24.30	33.90	48	29.40	26.00	34.00	0.221
BMI (kg/m^2^)	76	15.80	14.90	17.40	28	15.50	14.20	17.60	48	15.80	15.40	17.20	0.299
BMI-SDS-LMS	76	0.00	−0.70	0.60	28	−0.10	−1.00	0.90	48	0.00	−0.60	0.60	0.311
Height-SDS-LMS	76	0.40	0.10	1.10	28	0.40	−0.40	1.40	48	0.40	0.10	1.00	0.620
**Fitness parameters**													
VO2max (ml/kg/min)	76	49.00	44.30	53.30	28	46.50	42.60	49.50	48	50.30	47.20	53.90	**0.001**
Shuttle run rounds	76	30.50	20.00	43.00	28	30.00	20.00	41.80	48	32.50	18.30	47.00	0.829
Mean hand grip left (kg)	76	11.30	8.50	14.70	28	10.30	8.30	14.50	48	11.70	8.70	14.90	0.314
Mean hand grip right (kg)	76	12.70	9.30	15.50	28	11.50	9.00	14.30	48	13.80	9.30	16.00	0.116
**Cardiovascular parameters**													
Heart rate (bpm)	76	83.00	73.30	91.80	28	82.00	73.00	88.80	48	83.50	75.00	92.00	0.383
PSBP (mmHg)	76	114.00	104.00	118.00	28	112.50	102.00	118.00	48	114.00	104.00	118.00	0.838
PDBP (mmHg)	76	66.00	60.30	71.80	28	63.50	58.00	69.80	48	67.00	62.30	72.80	0.144
CSBP (mmHg)	76	96.00	89.00	102.50	28	96.50	87.50	103.00	48	95.50	89.30	101.00	0.897
CDBP (mmHg)	76	68.00	61.30	73.00	28	66.50	61.00	71.50	48	68.50	62.50	73.80	0.244
PWV (m/s)	76	4.50	4.20	4.70	28	4.50	4.10	4.80	48	4.60	4.20	4.70	0.991
BP systolic SDS (z) -Score	76	1.40	0.40	2.10	28	1.20	0.20	2.10	48	1.50	0.50	2.10	0.464
BP diastolic SDS (z) -Score	76	0.60	−0.30	1.40	28	0.20	−0.70	1.10	48	0.70	0.00	1.60	0.076
PWV age (z) -Score	53	1.40	0.20	2.50	20	1.00	−0.20	2.50	33	1.70	0.20	2.70	0.263
PWV height (z) -Score	70	0.90	−0.60	2.10	28	0.80	−0.40	2.20	42	1.10	−0.70	1.60	0.640
CSBP age (z) -Score	53	0.90	0.20	2.30	20	0.70	−0.40	2.10	33	0.90	0.30	2.40	0.378
CSBP height (z) -Score	70	0.60	−0.30	1.70	28	0.40	−0.40	1.80	42	0.60	−0.30	1.60	0.598

*P < 0.05 are shown in bold. BMI, body mass index; PSBP, peripheral systolic blood pressure; PDBP, peripheral diastolic blood pressure; CSBP, central systolic blood pressure; CDBP, central diastolic blood pressure; PWV, pulse wave velocity; BP, blood pressure*.

#### Age Groups

Table 2 presents anthropometric and cardiovascular data, CRF and hand grip strength testing results for the entire study population and according to age groups.

**Table 2:** Subject characteristics according to age groups.

	**Whole study group**				**Group 1: 6-7 years**				**Group 2: 8-9 years**				**Group 3: 10-11 years**							**Significance between groups**		
**Variables**	***N***	**Median**	**25th**	**75th**	***N***	**Median**	**25th**	**75th**	***N***	**Median**	**25th**	**75th**	***N***	**Median**	**25th**	**75th**	***X*^**2**^**	**DF**	***p*-value**	**1 vs. 2**	**1 vs. 3**	**2 vs. 3**
**Anthropometrics**																						
Age (years)	76	8.00	7.00	9.00	23	7.00	6.00	7.00	43	8.00	8.00	9.00	10	10.00	10.00	10.25	62.837	2	**<0.001**	**<0.001**	**<0.001**	**0.001**
Height (cm)	76	132.50	127.00	142.00	23	126.00	121.00	128.00	43	134.00	131.00	142.00	10	145.00	140.75	151.50	38.924	2	**<0.001**	**<0.001**	**<0.001**	**0.032**
Weight (kg)	76	28.00	25.00	34.00	23	25.00	23.00	27.00	43	30.00	26.10	34.00	10	36.00	31.33	40.50	22.759	2	**<0.001**	**<0.001**	**<0.001**	0.122
Body mass index (kg/m^2^)	76	15.80	14.90	17.40	23	15.87	14.11	17.16	43	15.75	14.92	17.12	10	17.32	15.39	18.02	1.605	2	0.448	**/**	**/**	**/**
BMI-SDS-LMS	76	0.00	−0.70	0.60	23	0.18	−0.92	0.76	43	−0.27	−0.76	0.73	10	0.20	−0.56	0.52	0.560	2	0.756	**/**	**/**	**/**
Height-SDS-LMS	76	0.40	0.10	1.10	23	0.65	0.09	1.03	43	0.40	−0.14	1.11	10	0.33	−0.25	0.95	0.546	2	0.761	/	/	/
**Fitness parameters**																						
VO2max (ml/kg/min)	76	49.00	44.30	53.30	23	50.51	42.45	53.49	43	48.13	44.29	53.17	10	50.77	48.51	53.00	10.938	2	**0.004**	0.088	**0.004**	1.000
Shuttle run rounds	76	30.50	20.00	43.00	23	20.00	16.00	30.00	43	38.00	25.00	47.00	10	31.50	21.75	49.50	10.362	2	**0.006**	0.168	**0.005**	1.000
Hand-grip left (kg)	76	11.30	8.50	14.70	23	8.33	5.33	11.00	43	12.33	10.00	15.00	10	15.17	13.00	17.75	21.770	2	**<0.001**	**0.001**	**<0.001**	0.251
Hand-grip right (kg)	76	12.70	9.30	15.50	23	9.00	7.33	11.00	43	14.00	11.00	16.00	10	14.83	13.92	17.00	24.113	2	<0.001	<0.001	<0.001	0.648
**Cardiovascular Parameters**																						
Heart rate (bpm)	76	83.00	73.30	91.80	23	83.00	75.00	88.00	43	83.00	73.00	91.00	10	90.50	72.25	93.75	0.702	2	0.704	**/**	**/**	**/**
PSBP (mmHg)	76	114.00	104.00	118.00	23	106.00	101.00	116.00	43	114.00	105.00	119.00	10	114.00	111.50	121.25	4.858	2	0.088	**/**	**/**	**/**
PDBP (mmHg)	76	66.00	60.30	71.80	23	64.00	58.00	69.00	43	66.00	60.00	73.00	10	67.50	61.00	72.50	1.035	2	0.596	**/**	**/**	**/**
CSBP (mmHg)	76	96.00	89.00	102.50	23	90.00	87.00	97.00	43	96.00	91.00	103.00	10	100.00	94.25	104.50	6.199	2	**0.045**	0.125	0.086	1.000
CDBP (mmHg)	76	68.00	61.30	73.00	23	66.00	58.00	70.00	43	68.00	62.00	75.00	10	69.50	63.25	74.50	2.474	2	0.290	**/**	**/**	**/**
PWV (m/s)	76	4.50	4.20	4.70	23	4.30	4.10	4.60	43	4.60	4.30	4.70	10	4.60	4.45	4.80	8.517	2	**0.014**	**0.030**	0.052	1.000
BP systolic SDS (z) -Score	76	1.40	0.40	2.10	23	0.90	0.19	2.08	43	1.59	0.52	2.12	10	1.16	0.51	1.61	2.345	2	0.310	**/**	**/**	**/**
BP diastolic SDS (z) -Score	76	0.60	−0.30	1.40	23	0.35	−0.48	1.29	43	0.67	−0.26	1.72	10	0.50	−0.40	1.36	0.172	2	0.918	**/**	**/**	**/**
PWV age (z) -Score	53	1.40	0.20	2.50	0	/	/	/	43	1.68	−0.04	2.92	10	0.79	0.25	1.84	1.990	1	0.158	**/**	**/**	**/**
PWV height (z) -Score	70	0.90	−0.60	2.10	18	0.83	−1.17	2.16	42	1.03	−0.52	2.10	10	0.78	−0.15	1.26	0.599	2	0.741	**/**	**/**	**/**
CSBD age (z) -Score	53	0.90	0.20	2.30	0	/	/	/	43	0.91	0.16	2.50	10	0.76	0.02	1.70	0.167	1	0.682	**/**	**/**	**/**
CSBD height (z) -Score	70	0.60	−0.30	1.70	18	0.89	−0.57	1.72	42	0.42	−0.33	1.73	10	0.53	−0.17	1.72	0.119	2	0.942	/	/	/

*P < 0.05 are shown in bold. DF, degrees of freedom; X^2^ Kruskal-Wallis chi-squared; BMI, body mass index; PSBP, peripheral systolic blood pressure; PDBP, peripheral diastolic blood pressure; CSBP, central systolic blood pressure; CDBP, central diastolic blood pressure; PWV, pulse wave velocity; BP, blood pressure*.

The-Kruskal-Wallis-H test showed that there was a statistically significant difference in CSBP [$X_{\left(2\right)}^{2}$ = 6.199, *p* = 0.045] and PWV [$X_{\left(2\right)}^{2}$ = 0.014, *p* = 0.030] between the different age groups (Table 3).

**Table 3:** Descriptive analysis of the studied population according to weight categories and blood pressure categories.

**Weight category**	**Girls**	**MV ± SD (kg)**	**Boys**	**MV ± SD (kg)**	**Total**	**MV ± SD (kg)**
Underweight (<10th percentile)	5	21.82 ± 4.12	3	24.40 ± 5.24	8 (10.5%)	22.79 ± 4.40
Normal weight (10th– ≤ 90th percentile)	19	29.39 ± 6.31	37	29.12 ± 4.60	56 (73.7%)	29.21 ± 5.19
Overweight (90th– ≤ 97th percentile)	4	34.98 ± 9.33	4	34.30 ± 6.35	8 (10.5%)	34.64 ± 7.39
Obese (>97th percentile)	0	–	4	37.48± 5.58	4 (5.3%)	37.48± 5.58
**Blood pressure category**	**Girls**	**MV** **±** **SD**	**Boys**	**MV** **±** **SD**	**Total**	**MV** **±** **SD**
Normotensive(<90th percentile)	13		18		31 (40.8%)	
BP sys SDS (z)-score <1.282 or		−1.52 ± 5.82		−0.20 ± 1.81		−0.76 ± 3.98
BP dia SDS (z)-score <1.282		−0.25 ± 0.72		−0.11 ± 0.98		−0.17 ± 0.87
High normotensive (90th−95th percentile)	3		7		10 (13.1%)	
BP sys SDS (z)-score ≥ 1.282, <1.645 or		1.58 ± 0.06		1.22 ± 0.39		1.33 ± 0.36
BP dia SDS (z)-score ≥ 1.282, <1.645		0.05 ± 1.14		0.28 ± 1.09		0.21 ± 1.05
Hypertensive (>95th percentile)	12		23		35 (46.1%)	
BP sys SDS (z)-score ≥1.645 or		2.36 ± 1.02		2.19 ± 0.48		2.25 ± 0.70
BP dia SDS (z)-score ≥1.645		1.03 ± 1.18		1.45 ± 0.87		1.31 ± 0.99

Further, they differed significantly in height [$X_{\left(2\right)}^{2}$ = 38.924, *p* = < 0.001] and weight [$X_{\left(2\right)}^{2}$ = 22.759, *p* < 0.001]. Moreover, groups revealed statistically significant differences in all fitness parameters, namely VO_2_max [$X_{\left(2\right)}^{2}$ = 10.938, *p* = 0.004], shuttle run rounds [$X_{\left(2\right)}^{2}$ = 10.362, *p* = 0.006], and hand-grip left [$X_{\left(2\right)}^{2}$ = 21.770, *p* < 0.001], and right [$X_{\left(2\right)}^{2}$ = 24.113, *p* < 0.001].

Follow-up tests were conducted to evaluate pairwise differences among the three groups, controlling for Type I error across test by using the Bonferroni approach. The results of these tests showed that there was a significant difference between age group 1 and 3 for VO_2_max (z = −2.178, *p* = 0.004, *r* = 0.556), shuttle run rounds (z = −1.911, *p* = 0.005, *r* = 0.552) and hand grip left (z = −3.745, *p* < 0.001, *r* = 0.724) and right (*z* = −4.063, *p* < 0.001, *r* = 0.707). The amount of hand grip force was also greater in group 2 than in group 1(left: z = −3.745, *p* = 0.001, *r* = 0.461; right: z = −4.276, *p* < 0.001, r = 0.526). Regarding cardiovascular parameters, group 1 only showed a significantly lower PWV compared to group 2 (z = −2.573, *p* = 0.030, *r* = 0.317) but not to the oldest age group. Further, the oldest children were significantly taller (compared to group 2: z = −2.548, *p* = 0.032, *r* = 0.350; compared to group 1: z = −5.670, *p* < 0.001, *r* = 0.987) and group 1 weighted significantly less than group 2 (z = −3.641, *p* < 0.001, *r* = 0.448) and 3 (z = −4.379, *p* < 0.001, *r* = 0.762). The strength of these relationship, as indexed by the effect size r, was always r >= 0.30 and therefore revealed medium to very large effects.

#### Cardiovascular Risk Factors

Table 3 depict weight and blood pressure categories and mean values +/− standard deviations divided by sex and in total.

Among all, 10.5% (5 girls/3 boys) were underweight, 73.7% (19 girls/37 boys) were normal weight, 10.5% (4 girls/4 boys) were overweight, and 5.3% (0 girls/4 boys) were obese (26). Almost half of the children, 46.1% (12 girls/23 boys), were classified as hypertensive, 13.1% (3 girls/7 boys) as high normotensive, and 40.8% (13 girls/18 boys) had a normal blood pressure according to criterion-reference (17).

As more than half of the study population presented with high normotensive or hypertensive blood pressure values, the population was divided into a healthy control group and a risk group for further analysis. The risk group consisted of all children with blood pressure classified as high normotensive and hypertensive or overweight and obese weight categories. The healthy control group only consisted of children with normal blood pressure and normal weight. Table 4 presents the characteristics of the sample stratified by risk factors.

**Table 4:** Subjects characteristics according to healthy group and group with risk factors.

	**Healthy group**				**Risk factors**				
**Variables**	***N***	**Median**	**25th**	**75th**	***N***	**Median**	**25th**	**75th**	***p*-value**
**Anthropometrics**									
Age (years)	24	8.00	7.00	9.00	49	8.00	7.00	9.00	0.966
Height (cm)	24	132.50	127.25	144.75	49	133.00	127.00	138.50	0.279
Weight (kg)	24	27.30	24.78	36.00	49	28.20	25.75	33.80	0.920
Body mass index (kg/m^2^)	24	15.91	14.84	17.08	49	15.87	14.99	18.42	0.428
BMI-SDS-LMS	24	−0.07	−0.71	0.42	49	0.05	−0.59	1.25	0.236
Height-SDS-LMS	24	0.66	0.30	1.43	49	0.28	−0.12	0.93	**0.014**
**Fitness parameters**									
VO2max (ml/kg/min)	24	48.61	44.05	52.62	49	49.54	44.14	53.24	0.511
Shuttle run rounds	24	29.00	17.75	42.25	49	35.00	20.00	43.50	0.372
Hand-grip left (kg)	24	10.83	8.08	14.67	49	11.33	9.33	14.83	0.573
Hand-grip right (kg)	24	11.50	8.83	14.83	49	13.33	9.33	15.83	0.466
**Cardiovascular parameters**									
Heart rate (bpm)	24	74.00	70.00	84.25	49	86.00	75.50	93.50	**0.002**
PSBP (mmHg)	24	102.50	97.50	109.00	49	116.00	113.50	119.50	**<0.001**
PDBP (mmHg)	24	60.50	58.00	65.50	49	69.00	62.50	73.50	**<0.001**
CSBP (mmHg)	24	89.00	84.00	94.50	49	99.00	94.00	104.00	**<0.001**
CDBP (mmHg)	24	62.50	60.00	67.75	49	70.00	66.00	75.50	**<0.001**
PWV (m/s)	24	4.20	4.03	4.50	49	4.60	4.50	4.70	**<0.001**
BP systolic SDS (z) -Score	24	0.18	−0.61	0.71	49	1.90	1.44	2.24	**<0.001**
BP diastolic SDS (z) -Score	24	−0.34	−0.70	0.35	49	1.12	0.01	1.85	**<0.001**
PWV age (z) -Score	15	−0.04	−0.38	1.07	36	2.22	1.00	3.06	**<0.001**
PWV height (z) -Score	22	−0.68	−1.29	0.25	45	1.44	0.92	2.27	**<0.001**
CSBD age (z) -Score	15	0.26	−1.03	0.68	36	1.60	0.53	2.51	**0.005**
CSBD height (z) -Score	22	−0.47	−1.33	0.06	45	1.58	0.25	1.85	**<0.001**

*P < 0.05 are shown in bold. Differences were testes using Mann-Whitney-U test. BMI. body mass index; PSBP. peripheral systolic blood pressure; PDBP. peripheral diastolic blood pressure; CSBP. central systolic blood pressure; CDBP. central diastolic blood pressure; PWV. pulse wave velocity; BP. blood pressure*.

The groups did not show significant differences in anthropometric and fitness status data. For cardiovascular data, several significant differences were observed.

The healthy control group revealed a lower heart rate (74.00 bpm (70.00;84.25) vs. 86.00 bpm (75.50;93.50), U = 319.0, *p* = 0.002, *r* = 0.363) and lower PWV (4.20 m/s (4.03;4.50) vs. 4.60 m/s (4.50;4.70), *U* = 230.5, *p* < 0.001, *r* = 0.490). As one inclusion criterion for the risk group was high to hypertensive blood pressure, the healthy group also presented with a lower PSBP (102.50 mmHg (97.50;109.00) vs. 116.00 mmHg (113.50;119.50), U = 111.0, *p* < 0.001, *r* = 0.643), PDBP (60.50 mmHg (58.00;65.50) vs. 69.00 (62.50;73.50), U = 261.0, *p* < 0.001, *r* = 0.441), CSBP (89.00 mmHg (84.00;94.50) vs. 99.00 mmHg (94.00;104.00), U = 251.0, *p* < 0.001, *r* = 0.454), and CDBP (62.50 mmHg (60.00;67.75) vs. 70 mmHg (66.00;75.50), U = 267.5, *p* < 0.001, *r* = 0.432). These differences were also detectable when CSBP was adjusted for age (0.26 (-1.03;0.68) vs. 1.60 (0.53;2.51), U = 133.0, *p* < 0.001, *r* = 0.397), and height (−0.47 (−1.33;0.06) vs. 1.58 (0.25;1.85), U = 154.0, *p* < 0.001, *r* = 0.556). Similar trends were discovered for age-adjusted PWV (−0.04 (−0.38;1.07) vs. 2.22 (1.00;3.06), U = 95.0, *p* < 0.001, *r* = 0.507), and height-adjusted PWV (−0.68 (−1.29;0.25) vs. 1.44 (0.92;2.27), U = 94.00, *p* < 0.001, *r* = 0.654).

#### Correlation Analysis

Among the whole study population, PWV showed a significant positive association with the anthropometrics age (*r* = 0.321, *p* = 0.005), height (*r* = 0.310, *p* = 0.006), and weight (*r* = 0.277, *p* = 0.015).

PWV z-scores adjusted to age as well as PWV z-scores adjusted to weight did not retain a significant association with shuttle run rounds, VO2max (ml/kg/min) or handgrip strengths. Corresponding results were found for CSBP (Table 5).

**Table 5:** Spearman correlation coefficients for A) PWV and B) CSBP.

	**Healthy subgroup**									**Risk subgroup**									**Whole study group**								
**A)**	**PWV (m/s)**			**PWV age (z)-score**			**PWV height (z)-score**			**PWV (m/s)**			**PWV age (z)-score**			**PWV height (z)-score**			**PWV (m/s)**			**PWV age (z)-score**			**PWV height (z)-score**		
**Variables**	***N***	***r***	***p***	***N***	***r***	***p***	***N***	***r***	***p***	***N***	***r***	***p***	***N***	***r***	***p***	***N***	***r***	***p***	***N***	***r***	***p***	***N***	***r***	***p***	***N***	***r***	***p***
Age (years)	24	0.679	**<0.001**	15	0.647	**0.009**	22	0.618	**0.002**	49	0.188	0.196	36	−0.327	0.051	45	−0.231	0.127	76	0.321	**0.005**	53	−0.198	0.156	70	−0.021	0.864
Sex	24	−0.012	0.955	15	0.248	0.373	22	−0.222	0.321	49	−0.118	0.418	36	0.097	0.575	45	−0.218	0.150	76	0.001	0.991	53	0.155	0.267	70	−0.056	0.643
Height (cm)	24	0.670	**<0.001**	15	0.728	**0.002**	22	0.493	**0.020**	49	0.274	0.057	36	0.051	0.770	45	−0.207	0.173	76	0.310	**0.006**	53	−0.022	0.874	70	−0.072	0.553
Weight (kg)	24	0.670	**<0.001**	15	0.782	**0.001**	22	0.562	**0.006**	49	0.078	0.592	36	−0.144	0.404	45	−0.172	0.260	76	0.277	**0.015**	53	−0.054	0.699	70	0.030	0.802
BMI (kg/m^2^)	24	0.488	**0.016**	15	0.533	**0.041**	22	0.456	**0.033**	49	−0.186	0.201	36	−0.215	0.208	45	−0.111	0.470	76	0.092	0.430	53	−0.064	0.647	70	0.097	0.423
Shuttle run rounds	24	−0.011	0.960	15	−0.306	0.267	22	−0.040	0.861	49	0.285	**0.047**	36	0.284	0.094	45	0.025	0.868	76	0.238	**0.038**	53	0.200	0.152	70	0.096	0.429
VO2max (ml/kg/min)	24	0.054	0.801	15	−0.129	0.647	22	−0.032	0.887	49	0.272	0.059	36	0.297	0.079	45	−0.040	0.796	76	0.225	0.050	53	0.233	0.093	70	0.038	0.757
Hand–grip left (kg)	24	0.479	**0.018**	15	0.627	**0.012**	22	0.320	0.146	49	0.224	0.122	36	0.059	0.734	45	−0.171	0.261	76	0.303	**0.008**	53	0.081	0.565	70	−0.008	0.947
Hand–grip right (kg)	24	0.502	**0.012**	15	0.578	**0.024**	22	0.369	0.091	49	0.248	0.086	36	0.093	0.590	45	−0.132	0.386	76	0.349	**0.002**	53	0.141	0.314	70	0.053	0.661
**B)**	**CSBP (mmHg)**			**CSBP age (z)–score**			**CSBP height (z)–score**			**CSBP (mmHg)**			**CSBP age (z)–score**			**CSBP height (z)–score**			**CSBP (mm Hg)**			**CSBP age (z)–score**			**CSBP height (z)–score**		
**Variables**	***N***	***r***	***p***	***N***	***r***	***p***	***N***	***r***	***p***	***N***	***r***	***p***	***N***	***r***	***p***	***N***	***r***	***p***	***N***	***r***	***p***	***N***	***r***	***p***	***N***	***r***	***p***
Age (years)	24	0.581	**0.003**	15	0.364	0.183	22	0.500	**0.018**	49	0.119	0.415	36	−0.247	0.146	45	−0.212	0.163	76	0.263	**0.022**	53	−0.114	0.417	70	−0.007	0.953
Sex	24	−0.036	0.866	15	0.341	0.214	22	−0.136	0.546	49	−0.149	0.305	36	−0.057	0.742	45	−0.297	**0.048**	76	−0.015	0.898	53	0.122	0.384	70	−0.064	0.601
Height (cm)	24	0.637	**0.001**	15	0.674	**0.006**	22	0.501	**0.018**	49	0.096	0.513	36	−0.189	0.269	45	−0.291	0.052	76	0.240	**0.037**	53	−0.054	0.702	70	−0.079	0.514
Weight (kg)	24	0.643	**0.001**	15	0.639	**0.010**	22	0.506	**0.016**	49	0.010	0.948	36	−0.257	0.130	45	−0.275	0.068	76	0.248	**0.030**	53	−0.078	0.579	70	−0.007	0.954
BMI (kg/m^2^)	24	0.436	**0.033**	15	0.283	0.307	22	0.307	0.165	49	−0.076	0.603	36	−0.203	0.235	45	−0.126	0.409	76	0.131	0.259	53	−0.086	0.540	70	0.067	0.581
Shuttle run rounds	24	−0.007	0.975	15	−0.215	0.441	22	−0.012	0.956	49	0.099	0.497	36	0.039	0.823	45	−0.032	0.837	76	0.150	**0.197**	53	0.023	0.871	70	0.031	0.802
VO2max (ml/kg/min)	24	0.055	0.799	15	0.013	0.965	22	0.050	0.824	49	0.091	0.536	36	0.048	0.780	45	−0.081	0.595	76	0.142	0.221	53	0.089	0.528	70	0.008	0.946
Hand–grip left (kg)	24	0.466	**0.022**	15	0.461	0.083	22	0.339	0.122	49	0.169	0.245	36	−0.037	0.832	45	−0.139	0.362	76	0.283	**0.013**	53	0.085	0.545	70	0.025	0.838
Hand–grip right (kg)	24	0.378	0.069	15	0.272	0.328	22	0.207	0.354	49	0.166	0.254	36	−0.051	0.768	45	−0.154	0.312	76	0.278	**0.015**	53	0.052	0.710	70	0.011	0.929

*PWV, pulse wave velocity; BMI, body mass index; CSBP, central systolic blood pressure. P < 0.05 are shown in bold*.

**Figure 2:**
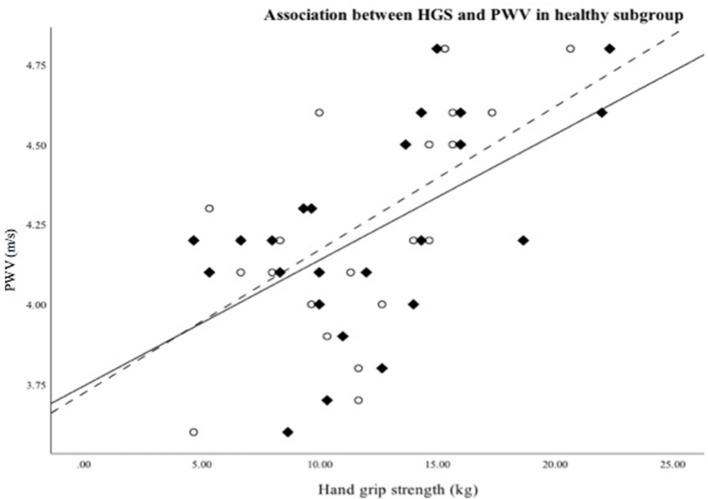
Scatterplot showing association between hand grip strength right (♦/—)/left (°/—) and PMW in healthy subgroup.

In the healthy control group PWV correlated significantly with anthropometric measures. Higher PWV was also associated with higher hand grip strength left and right (*r* = 0.470, *p* = 0.018; *r* = 0.502, *p* = 0.012). This association was still revealed when PWV values were transformed into age-specific (z)-scores (Figure 2) but did not reveal for PWV adjusted height (z)-scores. CSBP revealed similar correlations for anthropometrics. Only left hand grip strength showed a significant association with CSBP (*r* = 0.466, *p* = 0.022), but did not stay significant for the age and height adjusted z-scores (Table 5).

The risk group did not show significant relationships between the cardiovascular parameters (PWV and CSBP) and anthropometric measures, nor strength data. PWV only showed significant association with the amount of shuttle run rounds (*r* = 0.285, *p* = 0.047). CSBP transformed for height revealed a negative relationship with sex (*r* = −0.297, *p* = 0.048) (Table 5).

#### Regression Analysis

Multiple linear regression analysis was used to develop a model for predicting PWV and CSBP from participants' anthropometrics, fitness and strength status. However, for the whole study population no significant model could be found. Therefore, analyses were continued with the healthy subgroup only, which showed most of the significant associations in the correlation analyses.

In the healthy subgroup, a multiple linear regression was employed to predict PWV based on age, sex, cardiorespiratory and strength fitness. The results of the regression indicated that the model explained 38.8% (*R*^2^ = 0.388) of the variance. The model was a significant predictor of PWV [*F*_(6, 29)_ = 3.060, *p* = 0.019]. Nevertheless, none of the variables included in the regression analyses contributed significantly to the model (Table 6). Further, statistical analyses were continued with a multiple regression for predicting CSBP. The six predictor model was not significant [*F*_(6, 29)_ = 2.234, *p* = 0.068, *R*^2^ = 0.316).

**Table 6:** Multiple linear regression analysis.

	**(A) PWV**			**(B) CSBP**		
	**B ± SE**	**ß**	***p*-value**	**B ± SE**	**ß**	***p*-value**
Constant	4.438 ± 1.300		0.002	73.775 ± 37.397		0.058
Age (years)	0.108 ± 0.063	0.528	0.100	1.732 ± 1.820	0.312	0.349
Sex*	(−0.013) ± 0.113	−0.022	0.911	(−0.669) ± 3.254	−0.043	0.839
BMI (kg/m^2^)	(−0.003) ± 0.040	−0.016	0.938	0.388 ± 1.155	0.071	0.739
Shuttle run rounds (n)	0.004 ± 0.006	0.278	0.522	(−0.049) ± 0.183	−0.121	0.791
VO_2max_ (ml/kg/min)	(−0.026) ± 0.028	−0.472	0.362	(−0.134) ± 0.815	−0.089	0.871
Hand-grip strength (kg)*	0.014 ± 0.015	0.222	0.377	0.515 ± 0.443	0.303	0.255

P < 0.05. Dependent variable PVW, pulse wave velocity and CSBP, central systolic blood pressure. BMI, body mass index; Sex* Female=1, Male =2 in model; Hand-grip strength*: dominant hand.

## Discussion

Our key findings were that higher levels of CRF as well as higher levels of hand-grip strengths were not associated with improved vascular health or central systolic blood pressure in our sample of primary school children. Healthy children without overweight or hypertension reveal strong correlations between PWV age (z)-scores and anthropometric measures, such as height, weight and BMI. There were also no differences in CRF, number of shuttle run rounds or hand-grip strength between healthy children and children with cardiovascular risk factors. No sex differences in anthropometrics and cardiovascular risk factors could be found, however boys demonstrated higher VO2_max_ values than girls.

### Influence of Cardiovascular Risk Factors on Arterial Properties

In the present study, children classified as high normotensive or hypertensive and overweight or obese showed higher values for all arterial properties, namely heart rate, PBP, CBP, and PWV, compared to the healthy subgroup. This supports the idea that both obesity and hypertension at a young age can lead to an increase of arterial properties and therefore a greater risk for CVD (3, 8, 27). A longitudinal analysis in Finland, proved that individuals with high PWV in adulthood also had a higher prevalence of increased BP during childhood (27). Additionally, the importance of weight control in primary school children was also evident in the healthy subgroup, as BMI was positively associated with age and height adjusted PWV. These values are in good agreement with Pakhala et al. Cilek et al. and Sakuragi et al. and further support the role of weight reduction in young children (28–30). Also, the association between BMI and CSBP is in line with previous findings, where the authors highlight the positive correlation of increased BMI with CSBP in healthy children (*n* = 320, 14.0 ± 2.1 years) (5).

### Muscular Fitness and Arterial Properties

To the best of our knowledge, this study is the first to address the correlation between strength status and the arterial system among young German children. Notwithstanding many other studies have been done on strength status and vascular health, these were mainly tests with older individuals and reported inconsistent results (31, 32). Equally to the inconsistent findings in adults, the association between handgrip strength and arterial properties in childhood has shown contradictory results.

Cohen et al. tested n = 7329 youths from 10–17 years and demonstrated that in obese and unfit children good muscle strength may be a protective factor against high blood pressure (33). This inverse association supports findings by other authors who included not only blood pressure but also blood parameters to predict cardio-metabolic risk (34). On the other hand, Demmer et al. reported a positive correlation of handgrip strength with BP in 10,14, and 17 year olds (35). In a recent study by Zhang et al. participants aged 8–19 years (*n* = 2939) also showed positively associated BP with muscular fitness (36). These findings, along with ours, are opposite of what would be expected according to studies that show health benefits of resistance training in children (14, 37, 38). The underlying molecular mechanisms in young children regarding the effects of endurance and strength training are still unclear.

From healthy adults it is known that vascular endothelial cells play an important role in the regulation of vascular activity by producing vasoactive substances, such as endothelin-1 (ET-1) a vasoconstrictor and nitric oxide (NO) an endothelium-derived relaxing factor (39). Endurance training decreases the plasma concentration of ET-1. It is possible that changes in ET-1 and NO production caused by exercise training could promote differential changes in arterial stiffness. Alternatively, the increased arterial stiffness in relation to strength could be a result of a strong stimulus to increase sympathic nervous system activity, which may act to increase arterial stiffness by providing chronic restraint on the arterial wall via greater sympathetic adrenergic vasoconstrictor tone (40). Young children's running and play consist of acute changes in exercise intensity with acute intermittent elevations in arterial blood pressure. This may alter the arterial structure, or arterial load-bearing properties or both. These effects have been reported in a meta-analysis for resistance training in adults (41).

### Cardiorespiratory Fitness and Arterial Properties

As far as cardiorespiratory fitness parameters are concerned, we could not demonstrate that VO_2max_ was associated with stiffness parameters. Compared to the computed VO_2max_ values, the amount of shuttle run rounds were positively associated with PWV and CSBP in the whole study group, The results share similarities with Meyer et al. who studied surrogates of arterial stiffness in *n* = 646 German adolescents (age 13.9 ± 2.1 years) with the Mobil-O-Graph (9). In their cohort PWV was also positively associated with CRF, whereas CSBP showed no association. As mentioned earlier, Muller et al., demonstrated that BMI was a strong predictor for arterial stiffness. On top of that, they highlighted, that CRF was not the responsible determinant for vascular wall changes (5).

These findings are in contradiction with previous results reported in the literature (10, 30, 42). Looking at studies with similar age groups compared to our population, Sakuragi et al., Reed et al., and Weberruß et al., who also used the shuttle run test for assessing CRF, found inverse relationships between physical performance und arterial properties (30, 42, 43). This trend has been confirmed in a longitudinal analysis of pre-pubertal *n* = 44 obese children (8.9 ± 1.5 years) who performed a regular aerobic exercise training for 3-months (total 180 min/week in addition to physical education 135 min/week) (34).

The reason for our result is still not entirely clear, but differences might be explained partly by a bias through growth and maturity at this young age. In addition, it cannot be ruled out that the method of measuring CRF might have been influenced by external factors, for instance the motivation and compliance of the children to complete the shuttle run test.

### Study Strengths and Limitations

Strengths of the present study include the technique of measuring arterial parameters non-invasively. The oscillometric method is validated and provides acceptable accuracy compared to intra-aortic catheter measurements (19, 44) and can therefore be used as a standard in broad school settings. Further, the hand grip strength measurement is a non-invasive, and inexpensive method, which can be easily implemented and therefore used for further tests with children.

However, the present study must be interpreted within the context of its potential limitations. The study was performed in a cross sectional design, in a school setting, with a small sample size. We could implement three blood pressure measurements, however, we could not perform a 24-h BP measurement to confirm hypertension (Grade 1). This might be one confounder why nearly 46% of the study population showed hypertensive values (possibly white coat blood pressure elevation). Also the dietary intake of the young children was not evaluated. It is known that high dietary intake of salt has been linked to high blood pressure or hypertension. The hormonal status of the primary school children was not assessed. Although the work has limitations, we still believe it gives relevant information on determinants of vascular properties and could be a starting point for further investigations in strength-related arterial changes in young children.

## Conclusion

The results of the current study revealed that higher levels of CRF or higher levels of hand-grip strength is not associated to arterial stiffness and central systolic blood pressure in young children. Future research should reinforce the evaluation of underlying mechanisms concerning fitness and arterial elasticity and is required to understand the effects of physical activity in regard to CVD in young people.

## Data Availability Statement

The raw data supporting the conclusions of this article will be made available by the authors, without undue reservation, to any qualified researcher.

## Ethics Statement

The studies involving human participants were reviewed and approved by Technical University Munich, Faculty of Medicine (162/18S). Written informed consent to participate in this study was provided by the participants' legal guardian.

## Author Contributions

BB warrants that all aforementioned authors fulfill the criteria of authorship as defined by the International Committee of Medical Journal Editors (ICMJE). BB warrants that the work described in this manuscript has not been published before, that all authors approved the present submitted version and their institutions have no objections to the manuscript's contents.

## Conflict of Interest

The authors declare that the research was conducted in the absence of any commercial or financial relationships that could be construed as a potential conflict of interest.
